# Validation of a Modified Triage Scale in a Norwegian Pediatric Emergency Department

**DOI:** 10.1155/2018/4676758

**Published:** 2018-10-15

**Authors:** Mette Engan, Asle Hirth, Håvard Trønnes

**Affiliations:** Department of Pediatric and Adolescent Medicine, Haukeland University Hospital, 5021 Bergen, Norway

## Abstract

**Objective:**

Triage is a tool developed to identify patients who need immediate care and those who can safely wait. The aim of this study was to assess the validity and interrater reliability of a modified version of the pediatric South African triage scale (pSATS) in a single-center tertiary pediatric emergency department in Norway.

**Methods:**

This prospective, observational study included all patients with medical conditions, referred to the pediatric emergency department of a tertiary hospital in Norway from September 1, 2015, to November 17, 2015. Their assigned triage priority was compared with rate of hospitalization and resource utilization. Validity parameters were sensitivity, specificity, positive and negative predictive value, and percentage of over- and undertriage. Interrater agreement and accuracy of the triage ratings were calculated from triage performed by nurses on written case scenarios.

**Results:**

During the study period, 1171 patients arrived at the hospital for emergency assessment. A total of 790 patients (67 %) were triaged and included in the study. The percentage of hospital admission increased with increasing level of urgency, from 30 % of the patients triaged to priority green to 81 % of those triaged to priority red. The sensitivity was 74 %, the specificity was 48 %, the positive predictive value was 52 %, and the negative predictive value was 70 % for predicting hospitalization. The level of over- and undertriage was 52 % and 26 %, respectively. Resource utilization correlated with higher triage priority. The interrater agreement had an intraclass correlation coefficient of 0.99 by Cronbach's alpha, and the accuracy was 92 %.

**Conclusions:**

The modified pSATS had a moderate sensitivity and specificity but showed good correlation with resource utilization. The nurses demonstrated excellent interrater agreement and accuracy when triaging written case scenarios.

## 1. Introduction

Triage is a tool developed to identify the severely ill patients in a setting challenged with overcrowding and scarce resources [1–3]. In the emergency department (ED), triage aims to identify patients who need immediate care and those who can safely wait, in order to minimize mortality and morbidity [4].

Triage systems are algorithm based and prioritize patients into emergency levels using specific criteria for clinical urgency and relate this to recommended maximum waiting time. Initial versions of triage guidelines had three priority levels, but studies have argued that five-level triage systems are more effective, valid, and reliable [5, 6].

In Norway, Manchester Triage scale (MTS) and Rapid Emergency Triage and Treatment System Paediatric (RETTS-p) are five-level triage systems used in pediatric EDs. Other pediatric triage systems include the Canadian Paediatric Triage and Acuity Scale (paedCTAS), the Emergency Severity Index (ESI), the Australasian Triage Scale (ATS), and the South African triage scale (SATS).

The MTS involves 52 flow charts based on presenting complaints. General discriminators are then gathered after the patient is allocated to one of the charts. Some of the flow charts are specific for children [7, 8]. The ESI is based on four decision points regarding patient acuity and resource needs, in order to sort the patients into five triage levels. The RETTS-p combines presenting symptoms with measurements of vital parameters using emergency signs and symptoms cards. The paedCTAS use a list of presenting clinical complaints and symptoms including anamnestic parameters associated with high risk. The ATS provides criteria per urgency level and, like the MTS, RETTS-p, and paedCTAS, defines a time interval for review by a physician [8, 9].

The South African triage scale (SATS) is a nonlicensed and noncommercial triage system developed in 2004 for pre-and in-hospital emergency units throughout South Africa and contains an adult and a pediatric version [10–12]. It is a four-level triage scale consisting of clinical discriminators and a numeric triage early warning score (TEWS). The clinical discriminators aim to identify symptoms, signs, and anamnestic information, which require urgent attention even in the absence of abnormal vital signs. The TEWS is based on measurement of vital parameters organized in age specific TEWS tables. Vital signs are outside of the normal range for age and score points and add up to calculate the TEWS.

In 2012, a quality improvement group arranged at Haukeland University Hospital, Bergen, Norway, chose to implement SATS at the ED for adults. The triage system was selected because it provided a simple algorithm and seemed easy to perform and implement. In addition, it was noncommercial, and the group got permission to make adjustments to fit local conditions. SATS for adult patients was translated, modified, and implemented in the ED and in the prehospital emergency medical unit in 2013. A workgroup was then established to include the pediatric patients. The Western Norwegian regional health trust later implemented the complete triage for both adults and children in the EDs and for emergency ambulance transportations.

Ideally, a triage system should be reliable and valid with a high rate of interobserver agreement, good correlation with resource use requirements, and clinical outcomes prediction. Additionally, the triage process should be understandable and rapid to perform [6]. The evidence on the validity of pediatric triage systems remains insufficient, and there has been concern about the level of undertriage in several studies [13]. The SATS has been implemented and validated in low- and middle-income countries [10, 14–17], but only a few studies have investigated the performance of the pediatric SATS (pSATS) [18, 19]. This is the first study of a modified and translated version of the pSATS in a high-income country.

## 2. Methods

This validation study was conducted as part of the implementation of the pSATS at the Department of Pediatric and Adolescent Medicine at Haukeland University Hospital, Bergen, Norway.

To adapt the triage system to our pediatric population in a high-income country, a multidisciplinary triage work group of consultants, nurses, and paramedics adjusted the original flow chart and clinical discriminator list. The original TEWS tables were expanded from two into six different age categories and were modified using new data on age specific normal values for pulse and respiratory rate [20]. In addition, a score for pulse oximetry was added, and the score for injury was removed. A supplemental file provides an overview of the discriminator list and the TEWS tables (Figure S1).

The modified version of pSATS categorizes the children into triage priority red (emergency), orange (very urgent), yellow (urgent), green (not urgent), or blue (can wait). The triaging nurse has the opportunity to upgrade the triage priority using clinical judgement. The pediatrician should attend the child immediately if triaged to priority red, within 10 minutes to priority orange, within 60 minutes to priority yellow, and within 120 minutes to priority green. The priority blue was originally used for deceased patients arriving at the ED, but the triage work group decided to include patients arriving for administrative causes into this priority and thereby expended the triage to a 5-level scale. If possible, the children categorized to the blue priority should be attended within 120 minutes. For statistical analyses, the triage priorities were dichotomized into a high (red, orange, and yellow) or a low (green and blue) triage priority.

Children referred to the ED from the implementation day September 1, 2015, to November 17, 2015, were eligible. The study period was chosen to follow up the triage implementation. An electronic learning course and a detailed step-by-step user manual were developed by the triage work group to facilitate implementation and educate the nurses responsible for triage in the ED [21]. This manual complements the triage flow chart and provides information on how to perform the triage and indications for additional investigations.

The nurses categorized the patients into a triage priority using a paper triage form (supplemental file, Figure S1). The forms were scanned into the patients' electronic medical journal and then delivered for analysis. Descriptive data, diagnosis at discharge and information on resource utilization during the hospital stay, were collected retrospectively from the electronical medical journal. The diagnoses were categorized based on the ICD-10 system and in accordance with the Australian and New Zealand Paediatric Intensive Care diagnostic code table [22].

To determine interrater agreement and accuracy of the triage, 12 nurses working at the ED independently triaged 10 different written case scenarios.

## 3. Ethics Statement

The Data Protection Officer at Haukeland University hospital approved of the study and waived the requirement for consent. The regional ethical committee of Western Norway considered this study to be a quality assurance project and an application for ethical approval was not required (2017/543).

## 4. Study Design and Aims

This study has a prospective observational design and aims to assess the validity and interrater reliability of a modified version of the pSATS in a pediatric ED in Norway. The study was carried out in a tertiary hospital in Norway assessing only nonsurgical conditions.

The validity of a triage system is defined by how closely an acuity rating assigned using a triage scale is to the true acuity of the patient. However, limitations exist because of the lack of consensus of the reference standard for true urgency [3, 23, 24]. Surrogate markers, such as hospitalization, resource utilization, referral to intensive care unit, hospital stay, and expert's opinion, have been used to validate different triage systems [8, 24]. In the present study, we chose hospitalization as primary outcome and resource utilization as secondary outcome. Resources were defined as treatment with supplemental oxygen, intravenous (iv) antibiotics, or fluid therapy. Supplemental oxygen will usually be initiated at oxygen saturation level at 90-92 % or below. Fluid therapy must be prescribed by a physician and is most often given to patients with evidence of shock, moderate or severe dehydration, conditions that require fasting, and conditions with low fluid intake when nasogastric tube placement seems inappropriate. Iv antibiotics are usually initiated if there is a suspicion of severe bacterial infection and per oral treatment is assumed to be inadequate.

We aimed to explore differences in triage performance in different age groups and categorized the patients according to the age specific groups in the six TEWS tables. Since we expected few patients in the youngest and oldest age categories, we merged the age groups into 0-<1 year, 1 -<4 years, 4 -<7 years, and 7-14 years.

Validity parameters included sensitivity, specificity, positive and negative predictive value, and associated percentages for over- and undertriage. Sensitivity was the ability of the triage to correctly identify the patients that needed hospitalization, and, thus, the proportion of patient with a high triage priority among those admitted. Specificity was the ability of the triage to correctly identify the patients that did not need hospitalization, and, thus, the proportion of patients with a low triage priority among those not admitted. The positive predictive value was the proportion of patients admitted to hospital among those who were triaged to the high triage priority, and the negative predictive value was the proportion of patients not being admitted among those who were triaged to the low triage priority. Overtriage was defined as the proportion of children triaged into the high triage priority, who were not hospitalized. Conversely, undertriage was defined as the proportion of children triaged to the low triage priority, who were admitted to the ward.

## 5. Study Setting and Population

The Department of Pediatrics at Haukeland University hospital is a tertiary hospital and serves a population of approximately 100000 children aged 0-15 years. Every year, approximately 4500-5000 children attend the ED. Children are mainly referred by general practitioners who determine which patients require immediate care by a pediatrician. Some patients with presumed acute and serious conditions may also arrive directly by ambulance. Others were recently discharged from the Department of Pediatrics and had arrangements in place to return directly to the ED without consulting the primary care provider. Patients who were treated for surgical conditions or trauma were cared for in another ED, and the triage forms on those children were not a part of this study. Neonates referred from the maternity ward were assessed at the neonatal intensive care unit and did not undergo triage.

## 6. Data Analysis

The total number of referred patients in the study period was collected from the electronic patient journal system DIPS ASA 7.3.12.0. From this source, we also collected data on diagnoses and use of resources. Validity parameters were calculated using crosstabulations. Correlation between triage priority, hospitalization, and resource utilization was determined using Pearson chi-square test and binary logistic regressions. Interrater agreement for multiple raters was estimated using Cronbach's alpha intraclass correlation based on absolute agreement and a 2-way mixed-effects model. The accuracy was estimated by the percentage of agreement between the nurses' triage and a pSATS expert's rating. P-values < 0.05 were considered significant. Study sample size estimations were not performed. Sample size was determined by practical convenience and in line with other studies [19, 25–28]. Statistical analyses were performed using IBM SSPS statistics version 22.

## 7. Results

A total of 1171 children were referred to the Department of Pediatrics for emergency assessment during the study period, and 790 (67 %) patients were triaged and included in the study. Of those, 347 (44 %) were admitted to the ward and 443 (56 %) were treated as outpatients (supplemental file, Figure S2). The hospitalization rate was 58 % in the group who bypassed the triage and 48% in the total group of children. During the same time the previous year, the hospitalization rate was 49 %. We did not collect specific data on why the triage was not performed.

In our study, the mean age was 3.5 years (range 0-16 years and interquartile range 0.5-5 years) and slightly more boys (55 %) than girls were referred. Patients were categorized into diagnostic groups [22]. About 37 % had airway infections and 31 % had neurological, renal, or gastrointestinal disease (Table 1).

**Figure 1:**
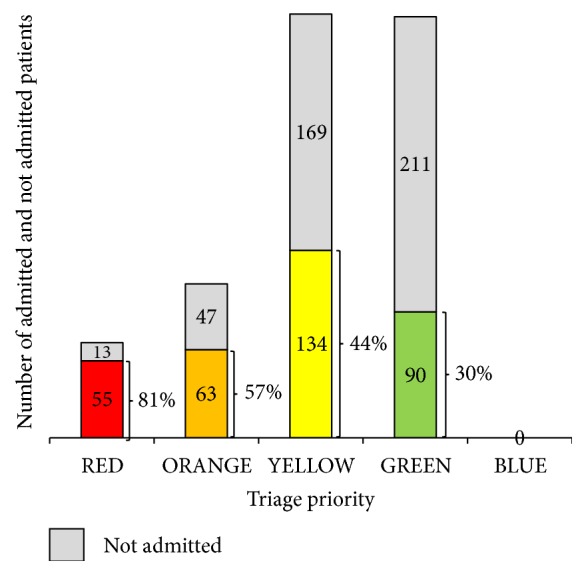
Number (N) of admitted and not admitted patients by the triage priority red, orange, yellow, green, and blue.

In the ED, 301 (38 %) children were triaged to priority green, 303 (39 %) to priority yellow, 110 (14 %) to priority orange, and 68 (9 %) to priority red. None were triaged to priority blue (Figure 1). The triage form was not completed on 8 patients, and these were excluded from further analysis. Hospitalization rate in triage priority green, yellow, orange, and red was 30 %, 44 %, 57 %, and 81 %, respectively (Figure 1). This progressive increase in hospitalization from the not urgent to the emergent triage priority was statistically significant (*X*^2^ (3, N = 782) = 69.2 p< 0.001).

Among the patients hospitalized, 74 % were triaged to the high triage priority (sensitivity) and 48 % of those who were not admitted were triaged to the low triage priority (specificity). Among patients triaged to the high triage priority, 52 % were hospitalized (PPV) and 70 % of those triaged to the low triage priority were not admitted (NPV). About 52 % of the patients triaged to the high triage priority were overtriaged, while about 26 % of those triaged to the low triage priority were undertriaged.

Further calculations were conducted after dividing the participants into four age groups (Tables 2 and 3). The sensitivity was increasing and the undertriage was decreasing for the children less than 4 years of age.

Compared to the priority green, the odds ratio (OR) for being hospitalized was 3.2 (95 % confidence interval (CI) 1.5-6.4), 5.3 (95 % CI 2.8-10.2), and 9.9 (95 % CI 5.2-19.1) for priority yellow, orange, and red, respectively (Table 4).

All 94 children receiving resources were hospitalized. This constituted 27 % of the admitted patients. A total of 11 patients received both iv antibiotics and iv fluid, and one patient received iv fluid, antibiotics, and supplemental oxygen. All 12 patients using two or more resource were categorized to priority red or orange in the ED. The OR for using one or more resources was 1.6 (95 % CI 0.8-3.3, p=0.214), 5.5 (95 % CI 2.7-11.1, p<0.001), and 5.2 (95 % CI 2.6-10.4, p<0.001) for priority yellow, orange, and red, respectively, compared to priority green.

The rate of agreement between the nurses triaging the written case scenarios had an intraclass correlation coefficient by Cronbach's alpha of 0.993 (95 % CI 0.985-0.998, p< 0.001). The percentage of exact agreement with an expert's opinion was 92 %. The misjudging was mainly about interpretation of level of consciousness.

## 8. Discussion

There is uncertainty about the applicability of medical guidelines developed in low- and middle-income countries to high-income countries, and vice versa [29]. This study provides new information about the validity of a modified version of pSATS in a high-income health care setting.

We found the modified pSATS to have a moderate sensitivity (74 %) and specificity (48 %). In 2011, Twomey M et al. studied the original pediatric triage at six different emergency centres in Western Cape, South Africa, and found it to be a robust triage tool for children with a sensitivity of 91 % and a specificity of 54.5 %, using hospital admission as a marker for urgency (Table 5) [19]. In comparison, we experienced a much higher rate of hospitalization (44 % versus 21.5 %), suggesting differences in health care organization and study populations.

In Botswana, the Accident and Emergency Department at the Princess Marina Hospital adapted the South African triage scale to create the PMH A&E Triage Scale (PATS). The validation study by Mullan et al. found an undertriage of 22 %, an overtriage of 29 %, and a corresponding sensitivity of 78 % for the pediatric subgroup, using hospitalization as primary outcome [18].

As expected, we observed an increased rate of hospitalization with increasing triage priority. In addition, patients triaged to a high priority required more resources. This is consistent with studies on other triage systems [13, 26, 30, 31]. The pSATS performed better on the youngest children. The oldest age group contained few participants, and the triage tool's validity on older children and adolescents should be further explored.

Ideally, a triage system should have no or minimal over- and undertriage. Overtriage will have minimal adverse consequences for the patients but may cause interruptions and can possibly wear out health care providers working in the ED. Undertriage is of more concern, as undertriaged patients may experience delays in treatment with potential harmful consequences. In the present study, there was an overtriage of 52 % and undertriage of 26 %. The level of undertriage was higher than previously reported [18, 19] and is of concern, since about one quarter of the hospitalized patients were assigned to the low triage priority.

The high level of undertriage in our study may have several reasons. It may be due to the referral routine, as the majority of the patients are preselected, being assessed, and referred by physicians in the primary health care. It is possible that some patients had already received effective treatment before arriving at the pediatric ED, diminishing the need for urgent attention, but still requiring admittance. In a high-income country, the threshold to hospitalize a patient may be lower than in low- and middle-income countries. In addition to the reasons mentioned above, we cannot discard the possibility that the modifications done to the original pSATS may have resulted in a tendency to undertriage patients. Future studies should investigate which vital signs and symptoms that most often correspond to hospitalization before making any changes to the triage form.

Several factors other than disease severity can influence the decision about admitting or discharging a patient. Haukeland University hospital serves a geographical wide area where the transport time to the hospital may be up to three hours by car. A decision of admission may be influenced by long transport time, especially if combined with late night hours or insecure or worried parents. Still, we experience that this accounts for only a small proportion of the admitted patients.

The study was not announced to the physicians working in the ED. However, introduction of the triage system itself may have influenced the decision-making process. The pSATS was not replacing another triage system, and the introduction to urgency levels and time limits to assess the patients may have affected the physicians' decision to admit a patient triaged to the urgent category. However, the triage was not announced to be a decision tool for hospitalization, and the hospitalization rate in the study group was slightly lower (44 %) compared to the hospitalization rate in the same period the year before (49 %).

Pediatric triage tools are difficult to compare because of variability in study design and population [32, 33]. This variation in assessment of validity has recently been reviewed by Kuriyama et al., who argue that reference standards, objective standard criteria for urgency, should be preferred in validation of scales [34]. A recent review by de Magalhães-Barbosa et al. summarizes the studies on MTS, paedCTAS, and ESI for pediatric emergency care. The triage tools performed lower outside the country where they were developed, and local validity and reliability studies are necessary. A consensus on study design, common reference standards, and considerations on cross-cultural adaption would be very advantageous [13]. So far, validation studies are hampered by lack of common reference standards and consequently the possibility for comparisons.

This study was based upon constructed validity, measuring hospitalization and resource utilization. Although hospitalization may on occasion be influenced by other conditions than disease severity, we believe hospitalization can serve as a surrogate marker for the “true” urgency level. This is supported by the finding of an increased rate of hospitalization with increasing triage priority. We believe the use of supplemental oxygen, iv fluid, and iv antibiotics are proper markers of disease urgency in our pediatric department, as these measures have restricted use in possible life-threatening conditions.

The interrater agreement is determined by the agreement in triage urgency priority if multiple nurses triage one patient or patient scenario [8]. A recent study on a pediatric triage system found a high degree of agreement between nurses prioritizing children in written case scenarios as well as in real life [35]. Another study comparing triage tool interrater reliability of live versus paper case scenarios concluded that there is moderate to high agreement between live cases and paper case scenarios and that the interrater reliabilities were acceptable in both cases [36]. This implies that the excellent interrater agreement and accuracy when triaging written case scenarios in this study may be applicable to triaging patients.

Strengths of the study include the relatively large number of patients included and a good interrater agreement. Nurses working in the ED performed the triage after basic training with excellent interrater agreement and accuracy. This may indicate that the modified pSATS was easy to understand and to carry out. The study provides new information on a triage tool used in several EDs and in the ambulance transport service in Norway. Despite only moderate sensitivity and specificity, we believe the implementation of the triage algorithm has contributed to a more systematic assessment of the patients and registration of vital parameters, in addition to classifying the level of urgency.

A major limitation to this study was that only 67 % of the eligible patients were triaged. The implementation date of the triage system in the ED coincided with the start of the study period, and, in retrospective, this was unfortunate. Most likely the failure of triaging all patients reflects difficulties in the implementation of this new routine. In cases when patients were admitted directly from the scheduled outpatient clinic, transferred from another department of the hospital, and referred during the night or attended by the physician before the triaging nurse, a triage was not always performed. This might be reflected in the somewhat lower hospitalization rate among the triaged patients (44 % versus 58 %), suggesting that triage was not performed if the decision on hospitalization was done in advance. We did not collect data on when or why the triage was not performed and are unable to say if the triage rate improved during the study period, or if this was a systematic bias possibly selecting the less urgent patients to the study.

Another limitation to this study is the lack of system for recording adverse events as consequences of over- and undertriage. We did not gather information on return to ED or whether the patients were treated in the primary health care after leaving the ED. We registered that none of the patients died.

The generalizability is limited to the selection of patients in a tertiary hospital, where children with surgical conditions and trauma were excluded. Further studies are necessary to explore the triage tool's performance on different diagnoses and in larger study populations. The reliability should be more thoroughly evaluated with real-life scenarios and both inter-rater and intrarater reliability analysis.

## 9. Conclusions

The modified pSATS evaluated in a single pediatric emergency department in a high-income country had a moderate sensitivity and specificity for predicting the need for hospital admission for medical conditions and showed good correlation with resource utilization. The level of undertriage was relatively high and of concern but may partly be related to national routines of referral and prehospital treatment. The nurses demonstrated excellent interrater agreement and accuracy when triaging written case scenarios. Although several validity parameters were lower than expected, we experienced that the implementation of the pSATS contributed to a more systematic assessment of the patients and greater awareness of the registration of vital parameters. We recommend the implementation of the pSATS in other pediatric emergency departments in Norway or other high-income countries, but with reservations that the medical staff should be aware of. The tool's performance on injured and surgical patients is still not evaluated, and we are concerned about the high level of undertriage.

## Figures and Tables

**Table 1:** Distribution of the diagnostic groups at discharge.

*Diagnostic groups at discharge*	*Total, n*	*(%)*	*Admitted, n *	*(%)*
Upper respiratory airway diseases	167	(21%)	45	(27%)
^1^Miscellaneous	134	(17%)	50	(37%)
^2^Lower respiratory airway diseases	125	(16 %)	70	(56%)
Gastrointestinal disease	112	(14 %)	59	(53%)
Other infections	111	(14 %)	47	(42%)
^3^Neurological disease	91	(12 %)	49	(54%)
^4^Renal disease	40	(5 %)	20	(50%)
^5^Injury	5	(0.6 %)	2	(40%)
Acquired cardiovascular disease	3	(0.4 %)	3	(100%)
Congenital cardiovascular disease	2	(0.3 %)	0	(0%)
Total	790	(100 %)	345	(44%)

Distribution of the different diagnostic groups at discharge by number (n) and percent (%) for the total study group and for the admitted group [22].

^1^ Miscellaneous including dehydration, diabetes ketoacidosis, and cancer.

^2^ Lower respiratory airway diseases including asthma, bronchiolitis, pneumonia, or pneumonitis.

^3^ Neurological disease including seizures and meningitis.

^4^ Renal disease including urinary tract infection.

^5^ Injury including only concussion of the brain and nonaccidental trauma.

**Table 2:** The distribution of assigned triage priority by age groups.

Age, *years*	Red	Orange	Yellow	Green	Total
	*n(a)*	*n(a)*	*n(a)*	*n(a)*	*N(a)*
0 - < 1	29 (23)	15 (14)	125 (59)	87 (24)	256 (120)
1 - < 4	25 (18)	51 (25)	97 (36)	103 (24)	276 (103)
4 - < 7	14 (14)	38 (23)	67 (29)	91 (30)	210 (96)
7- 14	0	6 (1)	14 (10)	20 (12)	40 (23)
Total	68 (55)	110 (63)	303 (134)	301 (90)	782 (342)

Number (n) of patients by age groups and triage priority. The number of patients admitted (a) to ward is shown in brackets.

**Table 3:** Sensitivity, specificity, over- and, undertriage by age groups.

Age	Sensitivity	Specificity	Overtriage	Undertriage	Pearson chi-square test		
*years*	*%*	*%*	*%*	*%*	*X* ^*2*^		*p-value*
0 - < 1	80	46	54	20	(3, N = 256)	= 38.3	p<0.001
1 - < 4	77	46	54	23	(3, N = 276)	= 24.5	p<0.001
4 - < 7	69	54	46	31	(3, N = 210)	= 26.1	p<0.001
7 - 14	48	47	53	52	(2, N = 40)	= 5.3	p= 0.07
Total	74	48	52	26	(3, N = 782)	= 69.8	p<0.001

**Table 4:** Odds ratio for hospitalization according to triage priority.

**Triage priority**	OR (95 % CI)	p-value
Red	9.9 (5.2-19.1)	<0.001
Orange	5.3 (2.8-10.2)	<0.001
Yellow	3.2 (1.5-6.4)	0.002
Green	Reference category	

The odds ratio (OR) reported with 95 % confidence interval (CI) of being hospitalized compared to the not urgent priority green.

**Table 5:** Comparisonof data from the study on the original pSATS [19] and the modified pSATS.

	Original pSATS [19]	Modified pSATS
Number of childre in ED (n)	2014	790
Admitted to ward (%)	21.5	44
Sensitivity (%)	91	74
Specificity (%)	54.5	48
Undertriage (%)	9	26
Overtriage (%)	45.5	52

## Data Availability

The data used to support the findings of this study are included within the Supplementary Material file.

## Abbreviations

ATS: Australasian Triage System
AVPU: Alert, Voice, Pain, Unresponsive
CI: Confidence interval
ED: Emergency department
ESI: Emergency Severity Index
Iv: Intravenous
MTS: Manchester Triage Scale
N: Number
NPV: Negative predictive value
OR: Odds ratio
paedCTAS: Canadian Paediatric Triage and Acuity Scale
PPV: Positive predictive value
pSATS: Pediatric South African triage scale
RETTS-p: Rapid Emergency Triage and Treatment System Paediatric
SATS: South African triage scale
TEWS: Triage early warning score

## Supplementary Materials

Supplementary Material
** Figure S1: The pSATS triage form. **The triage form is translated by the first author and contains the modified discriminator list and TEWS tables for the six age groups. The Norwegian triage form contains supplemental footnotes to elaborate some of the discriminators. These are available in the English translated version of the manual at https://helse-bergen.no/seksjon/mottaksklinikken/Documents/SATS-N%20users%20manual%20version%203.02.pdf (PDF, 2 pages, 275 KB).** Figure S2: **Overview of the recruitment of patients in the emergency department (ED) by number.** Tables S1-S5: **Additional descriptive data on the collected material.
