# Supplementary material for: Validation of a Modified Triage Scale in a Norwegian Pediatric Emergency Department
Source: Int J Pediatr. 2018 Oct 15;2018:4676758. doi: 10.1155/2018/4676758 (PMC6205310; doi:10.1155/2018/4676758)
Supplement: Supplementary Material — Figure S1: The pSATS triage form. The triage form is translated by the first author and contains the modified discriminator list and TEWS tables for the six age groups. The Norwegian triage form contains supplemental footnotes to elaborate some of the discriminators. These are available in the English translated version of the manual at https://helse-bergen.no/seksjon/mottaksklinikken/Documents/SATS-N%20users%20manual%20version%203.02.pdf (PDF, 2 pages, 275 KB). Figure S2: Overview of the recruitment of patients in the emergency department (ED) by number. Tables S1-S5: Additional descriptive data on the collected material. [file 4676758.f1.pdf]

ID: [ ]

Date: ________ Time: ________

Prehospital triage ☐ ☐ ☐ ☐ ☐

**Trauma** □ Yes □ No

**1. Discriminator list**

No match: ☐

| RED PRIORITY -EMERGENCY- | ORANGE PRIORITY -VERY URGENT- | YELLOW PRIORITY -URGENT- |
|---|---|---|
| | Allergic reaction, acute | Baby < 2 month |
| | Battery swallowed | |
| Unconscious patient | Consciousness, reduced/lethargic | |
| Haemorrhage, major | | |
| Burn, face/inhalation/circumferential , > 9% or high voltage | | Burn: minor |
| Fracture with suspected vascular injury | Fracture, compound or displaced, or dislocated joint | Fractur, closed or ankle fracture < 8 h. since trauma |
| Cyanosis, central (SpO2 < 90 %) | Chest pain, current | |
| Downing | Dehydration, no urine past 12 h. | Dehydration, little urine last 24 h. |
| Fever and neutropenic, immunodeficient or baby < 3 month | Intoxication or poisoning | |
| Cardiac arrest/CPR | Headache, acute and intense | |
| Head injury, severe GCS under 9 | Head injury, moderate GCS 9-13 | Head injury, minor GCS 14-15 |
| Hypoglycemia, blood glucose < 3 mmol/L | Hyperglycaemia, blood glucose > 11 mmol/L and shortness of breath | |
| Convulsions, current | Neurological deficits, acute | Convulsions, now alert |
| Airway, airway at risk, intubated or foreign body | Vomiting, fresh blood or bile-coloured | Vomiting or diarrhoea, persistent |
| Scrotum, acute and severe pain | Pain, acute severe or inconsolable crying | Pain, moderate |
| Trauma, seriously injured patient | | |
| Shortness of breath, very laboured, obstructive or apnoea | Shortness of breath, moderate laboured/obstructive | Shortness of breath, somewhat laboured or obstructive |
| | Eye injury, caustic or penetrating | |

**2. TEWS - Triage early warning score**

| | | | | | | | | | | | | | |
|---|---|---|---|---|---|---|---|---|---|---|---|---|---|
| **Time** | | **Height** | | **Weight** | | **Head circum.** | | **BP** | | **GCS** | | **Glucose** | |
| **Tp** | | **RR** | | **SpO₂** | | **Pulse** | | **Cap.re** | | **AVPU** | | **TEWS** | |

Optionally upgrade by clinical judgement: **≥ 7** **5-6** **3-4** **0-2**

## 3. TRIAGE PRIORITY LEVEL

☐ ☐ ☐ ☐ ☐

**Nurse sign.** __________ **Time** __________

**Physician sign.** __________ **Time** ________

| TEWS<br>Newborn < 1 month | 3 | 2 | 1 | 0 | 1 | 2 | 3 |
|---|---|---|---|---|---|---|---|
| **Respiratory rate** | < 25 | | 25 - 39 | 40 - 55 | 56 - 64 | 65 - 79 | ≥ 80 |
| **$SpO_2$** | < 90 % with $O_2$ | > 90 % with $O_2$ | 90 - 95 % without $O_2$ | > 95 % | | | |
| **Heart rate** | < 85 | | 85 - 99 | 100 - 160 | 161 - 169 | 170 - 189 | ≥ 190 |
| **Capillary refill time** | | | | 1 - 2 sec | 3 sec. | | ≥ 4 sec. |
| **AVPU** | | | | **A:** Awake and alert | **V:** Reacts to voice | **P**: Reacts to pain | **U:** Unresponsive |
| **Temperature** | | Feels cold or < 36° | | 36°- 38° | | ≥ 38.1° | |
| **Mobility** | | | | Normal for age | | Unable to move as normal | |

| TEWS<br>Child 1-12 month | 3 | 2 | 1 | 0 | 1 | 2 | 3 |
|---|---|---|---|---|---|---|---|
| **Respiratory rate** | < 20 | | 20 - 34 | 35 - 45 | 46 - 54 | 55 - 69 | ≥ 70 |
| **$SpO_2$** | < 90 % with $O_2$ | > 90 % with $O_2$ | 90 - 95 % without $O_2$ | > 95 % | | | |
| **Heart rate** | < 80 | | 80 - 99 | 100 - 160 | 161 - 169 | 170 - 189 | ≥ 190 |
| **Capillary refill time** | | | | 1 - 2 sec | 3 sec. | | ≥ 4 sec. |
| **AVPU** | | | | **A:** Awake and alert | **V:** Reacts to voice | **P**: Reacts to pain | **U:** Unresponsive |
| **Temperature** | | Feels cold or < 36° | | 36°- 38° | 38.1°- 39° | ≥ 39.1° | |
| **Mobility** | | | | Normal for age | | Unable to move as normal | |

| TEWS<br>Child 1-3 years | 3 | 2 | 1 | 0 | 1 | 2 | 3 |
|---|---|---|---|---|---|---|---|
| **Respiratory rate** | < 20 | | 20 - 24 | 25 - 35 | 36 - 44 | 45 - 59 | ≥ 60 |
| **$SpO_2$** | < 90 % with $O_2$ | > 90 % with $O_2$ | 90 - 95 % without $O_2$ | > 95 % | | | |
| **Heart rate** | < 70 | | 70 - 89 | 90 - 130 | 131 - 139 | 140 - 159 | ≥ 160 |
| **Capillary refill time** | | | | 1 - 2 sec | 3 sec. | | ≥ 4 sec. |
| **AVPU** | | Acute confusion | | **A:** Awake and alert | **V:** Reacts to voice | **P**: Reacts to pain | **U:** Unresponsive |
| **Temperature** | | Feels cold or < 36° | | 36°- 38° | 38.1° - 39° | ≥ 39.1° | |
| **Mobility** | | | | Normal for age | | Unable to move as normal | |

| **TEWS Child 4-6 years** | **3** | **2** | **1** | **0** | **1** | **2** | **3** |
|---|---|---|---|---|---|---|---|
| **Respiratory rate** | < 15 | | 15 - 19 | 20 - 24 | 25 - 29 | 30 - 44 | ≥ 45 |
| **$SpO_2$** | < 90 % with $O_2$ | > 90 % with $O_2$ | 90 - 95 % without $O_2$ | > 95 % | | | |
| **Heart rate** | < 60 | | 60 - 69 | 70 - 120 | 121 - 129 | 130 - 149 | ≥ 150 |
| **Capillary refill time** | | | | 1 - 2 sec | 3 sec. | | ≥ 4 sec. |
| **AVPU** | | Acute confusion | | **A:** Awake and alert | **V:** Reacts to voice | **P**: Reacts to pain | **U:** Unresponsive |
| **Temperature** | | Feels cold or < 36° | | 36°- 38° | 38.1°- 39° | ≥ 39.1° | |
| **Mobility** | | | | Normal for age | | Unable to move as normal | |

| **TEWS Child 7-12 years** | **3** | **2** | **1** | **0** | **1** | **2** | **3** |
|---|---|---|---|---|---|---|---|
| **Respiratory rate** | < 14 | | 14 - 18 | 19 - 22 | 23 - 29 | 30 - 39 | ≥ 40 |
| **$SpO_2$** | < 90 % with $O_2$ | > 90 % with $O_2$ | 90 - 95 % without $O_2$ | > 95 % | | | |
| **Heart rate** | < 60 | | 60 - 69 | 70 - 110 | 111 - 119 | 120 - 139 | ≥ 140 |
| **Capillary refill time** | | | | 1 - 2 sec | 3 sec. | | ≥ 4 sec. |
| **AVPU** | | Acute confusion | | **A:** Awake and alert | **V:** Reacts to voice | **P**: Reacts to pain | **U:** Unresponsive |
| **Temperature** | | Feels cold or < 36° | | 36°- 38° | 38.1° - 39° | ≥ 39.1° | |
| **Mobility** | | | | Normal for age | | Unable to move as normal | |

| **TEWS Youth 13-14 years** | **3** | **2** | **1** | **0** | **1** | **2** | **3** |
|---|---|---|---|---|---|---|---|
| **Respiratory rate** | < 9 | | 9 - 13 | 14 - 19 | | 20 - 29 | ≥ 30 |
| **$SpO_2$** | < 90 % with $O_2$ | > 90 % with $O_2$ | 90 - 95 % without $O_2$ | > 95 % | | | |
| **Heart rate** | < 45 | | 45 - 54 | 55 - 95 | 96 - 114 | 115 - 129 | ≥ 130 |
| **Capillary refill time** | ≤ 70 | 71 - 80 | 81 - 100 | 101 - 180 | | ≥ 180 | |
| **AVPU** | | Acute confusion | | **A:** Awake and alert | **V:** Reacts to voice | **P**: Reacts to pain | **U:** Unresponsive |
| **Temperature** | | Feels cold or < 36° | | 36°- 38° | 38.1°- 39° | ≥ 39.1° | |
| **Mobility** | | | | Normal for age | | Unable to move as normal | |

**Supplemental material**

**Figure S2.** Overview of the recruitment of patients in the emergency department (ED) by number.

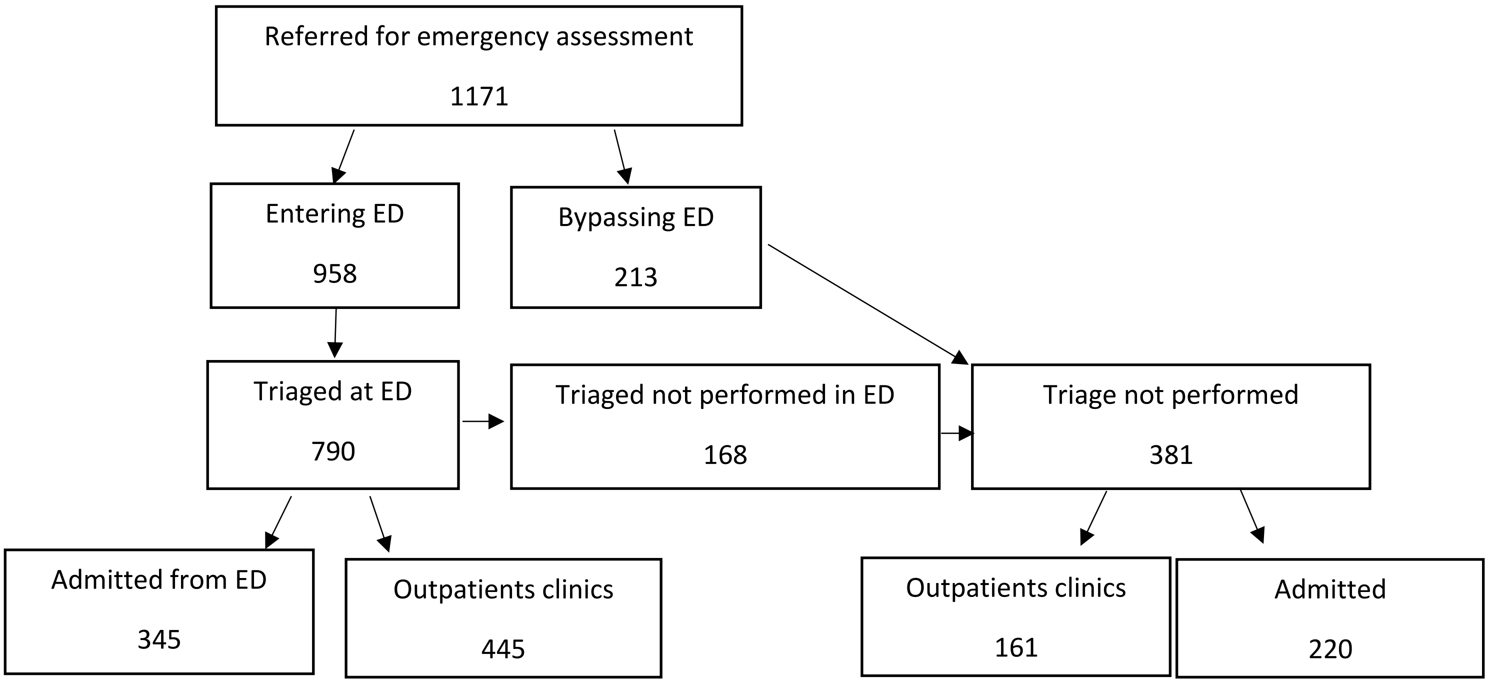

**Supplemental material**

**Table S1** Overview of the patients categorized into the non-urgent triage priority green and the distribution of diagnosis at discharge compared to all of the included patients.

| Diagnosis | Green admitted to ward | Green discharged from ED | Green total | All patients | All admitted patients |
|---|---|---|---|---|---|
| | n (%) | n | N (%) | % | % |
| Upper respiratory airway diseases | 7(11%) | 54 | 61(20%) | 21% | 27% |
| Lower respiratory airway diseases | 5(19%) | 21 | 26(9%) | 16% | 56% |
| Gastrointestinal disease | 16(36%) | 28 | 44(15%) | 14% | 53% |
| Renal diseases | 6(33%) | 12 | 18(6%) | 5% | 50% |
| Other infections | 19(34%) | 37 | 56(19%) | 14% | 42% |
| Miscellaneous | 26(37%) | 44 | 70(23%) | 17% | 37% |
| Neurological disease | 11(48%) | 12 | 23(8%) | 12% | 54% |
| Cardiovascular disease | 0(0%) | 1 | 1(0.3%) | 0.7% | 60% |
| Injury | 0(0) | 2 | 2(0.7%) | 0.6% | 40% |
| **Total** | 90(30%) | 211 | 301(100%) | 100% | 44% |

**Table S2** Overview of the patients categorized into the urgent triage priority yellow and the distribution of diagnosis at discharge compared to all of the included patients. The internal distribution of triage early warning score (TEWS) in the yellow priority group.

| Diagnosis | Yellow admitted to ward | Yellow discharged from ED | Yellow total | All patients | All admitted patients |
|---|---|---|---|---|---|
| | n (%) | n | N (%) | % | % |
| Upper respiratory airway diseases | 21(32%) | 44 | 65(21%) | 21% | 27% |
| Lower respiratory airway diseases | 32(56%) | 25 | 57(19%) | 16% | 56% |
| Gastrointestinal disease | 30(62%) | 22 | 52(17%) | 14% | 53% |
| Renal diseases | 8(53%) | 7 | 15(5%) | 5% | 50% |
| Other infections | 11(34%) | 21 | 32(11%) | 14% | 42% |
| Miscellaneous | 14(37%) | 24 | 38(13%) | 17% | 37% |
| Neurological disease | 15(38%) | 24 | 39(13%) | 12% | 54% |
| Cardiovascular disease | 2(67%) | 1 | 3(1%) | 0.7% | 60% |
| Injury | 1(50%) | 1 | 2(0.7%) | 0.6% | 40% |
| **Total** | 169(44%) | 169 | 303(100%) | 100% | 44% |
| | | | | | |
| **TEWS 0-1-2** | 73 | 86 | 159 | | |
| **TEWS 3-4** | 56 | 80 | 136 | | |

**Supplemental material**

**Table S3** Descriptive data on triage priority and recourses used

| **Triage priority** | **Number of resources used** | | | | **% use of any resource** |
|---|---|---|---|---|---|
| | **0** | **1** | **2** | **3** | |
| Red | 44 | 17 | 6 | 1 | 35% |
| Orange | 85 | 22 | 3 | 0 | 23% |
| Yellow | 281 | 21 | 1 | 0 | 7% |
| Green | 278 | 22 | 1 | 0 | 8% |
| Total | 696 | 82 | 11 | 1 | 12% |

**Table S4** Comparing the odds ratio of being hospitalized or using resources compared to priority green. The calculations are based om binary logistic regression with *hospitalization* or the need for *one or more resources* as dependent variable and triage level as categorical covariate, priority green set to reference category.

**Odds ratio for hospitalization according to triage priority**

| *Triage priority* | *Odds ratio (95% Confidence interval)* | *p-value* |
|---|---|---|
| Red | 9.9 (5.2-19.1) | <0.001 |
| Orange | 5.3 (2.8-10.2) | <0.001 |
| Yellow | 3.2 (1.5-6.4) | 0.002 |
| Green | Reference category | - |
| Blue | - | - |

**Odds ratio for resource utilization according to triage priority**

| *Triage priority* | *Odds ratio (95% Confidence interval)* | *p-value* |
|---|---|---|
| Red | 5.2 (2.6-10.4) | <0.001 |
| Orange | 5.5 (2.7-11.1) | <0.001 |
| Yellow | 1.6 (0.8-3.3) | 0.214 |
| Green | Reference category | - |
| Blue | - | - |

**Supplemental material**

## Table S5 Reliability study

Percentage of exact agreement with expert's opinion was 92%. Intraclass correlation coefficient by Cronbach's alpha of 0.993 (95 % CI 0.985-0.998, $p < 0.001$).

| Nurse 1 | Nurse 2 | Nurse 3 | Nurse 4 | Nurse 5 | Nurse 6 | Nurse 7 | Nurse 8 | Nurse 9 | Nurse 10 | Nurse 11 | Nurse 12 | Expert . |
|---|---|---|---|---|---|---|---|---|---|---|---|---|
| 2 | 2 | 2 | 2 | 2 | 2 | 2 | 2 | 2 | 2 | 2 | 2 | 2 |
| 1 | 1 | 1 | 1 | 1 | 1 | 1 | 2 | 1 | 1 | 1 | 1 | 1 |
| 2 | 2 | 2 | 2 | 2 | 2 | 2 | 2 | 2 | 2 | 2 | 2 | 2 |
| 3 | 2 | 3 | 2 | 3 | 2 | 2 | 3 | 2 | 3 | 3 | 2 | 2 |
| 2 | 2 | 2 | 2 | 2 | 2 | 2 | 2 | 2 | 2 | 2 | 2 | 2 |
| 2 | 2 | 2 | 2 | 2 | 2 | 2 | 2 | 2 | 1 | 2 | 2 | 2 |
| 4 | 4 | 4 | 4 | 4 | 4 | 4 | 4 | 4 | 4 | 4 | 4 | 4 |
| 2 | 2 | 2 | 2 | 2 | 2 | 2 | 2 | 2 | 2 | 2 | 2 | 2 |
| 1 | 1 | 1 | 1 | 1 | 1 | 1 | 2 | 1 | 2 | 1 | 1 | 1 |
| 3 | 3 | 3 | 3 | 3 | 3 | 3 | 3 | 3 | 3 | 3 | 3 | 3 |
